# Social anxiety and celebrity worship: the mediating effects of mobile phone dependence and moderating effects of family socioeconomic status

**DOI:** 10.1186/s40359-023-01405-x

**Published:** 2023-10-31

**Authors:** Rong Jia, Qing Yang, Bo Liu, Han Song, Zhengjun Wang

**Affiliations:** 1 School of Psychology, Shaanxi Normal University, 199 South Chang’an Road, Xi’an, 710062 China; 2 Yuncheng Central Hospital of Shanxi Province, Yuncheng, China; 3 College of Education, Hebei Normal University, Shijiazhuang, China

**Keywords:** Celebrity worship, Social anxiety, Socioeconomic status, Mobile phone dependence, Moderated mediation model

## Abstract

The Absorption-addiction model suggests that people worship celebrities to compensate for some personal or social defects, so poor mental state is related to celebrity worship. The current study aimed to explore the underlying mechanisms influencing celebrity worship. A total of 1,147 participants (aged 19–26 years) completed online questionnaires to assess social anxiety, mobile phone dependence, parental income and celebrity worship. Results showed that: (1) social anxiety, socioeconomic status (SES) and celebrity worship were positively correlated; (2) Social anxiety affected celebrity worship through mobile phone dependence; (3) SES played a moderating role in the mediation model. At higher levels of SES, individuals with high social anxiety showed reduced dependence on mobile phones. These findings highlight the importance of mobile phone dependence and family SES in celebrity worship. Additionally, the findings demonstrated that females are more likely to pay attention to celebrities, but the greater SES and reduced mobile phone dependence can mitigate their celebrity addiction.

## Introduction

The popularity of the Internet makes it easier for individuals to participate in parasocial relationships [1], with celebrities, and more young people worshipping idols than in previous generations. The parasocial relationship is a one-sided emotional bond between a fan and a celebrity [2–4]. Celebrity worship can be considered to be a continuum ranging from enthusiastic concern for the celebrity’s health to compulsive behaviors to a pathological obsession with a favorite celebrity, usually measured by the Celebrity Atitude Scale (CAS) [2, 3, 5]. Recent studies have reported a positive relationship between celebrity worship and behavioral addictions such as gambling addiction [6], problematic Internet use [5], psychoactive substance use [7] and problematic social media use [8]. Many studies have also shown that high levels of celebrity worship are associated with a range of adverse psychological states, such as anxiety, depression [9], and obsessive thoughts [10].

However, most of those studies are conducted in Western contexts, and only a few studies have investigated the status quo of celebrity worship in China, which mostly focused on the adolescents [11]. As mentioned in previous study [12], though the percentage of celebrity worship shows a decreasing tendency from junior high school to university, nearly half of college students (45.3%) report that they have a favorite celebrity. It is still necessary to investigate the celebrity worship of college students. Thus, based on the college students in China, the present study aims to explore the effect of social anxiety on celebrity worship, as well as the mediating role of mobile phone addiction and the moderating role of socioeconomic status.

### Social anxiety and celebrity worship

In this study, social anxiety was defined as anxiety caused by individuals in real or imagined social situations [13]. According to the Absorption-Addiction model, celebrity worship compensates for what the worshipper lacks, such as meaningful interpersonal relationships, a sense of emptiness and/or a stable identity [3, 14, 15]. Maltby et al. [9] reported that celebrity worship was positively related to anxiety and social dysfunction, but negatively related to loneliness [16] and cognitive skills [17]. However, the relationship between social anxiety and celebrity worship remains unknown. The social compensation hypothesis suggests that individuals with high levels of social anxiety are more likely to compensate for feelings of loneliness and emptiness through mobile phone use, online games, and by focusing on celebrities [18, 19]. Also, previous studies have shown a positive correlation between social anxiety and parasocial relationships [20, 21]. Therefore, social anxiety may be a predictor of parasocial relationships between fans and celebrities. Besides, most studies found no gender differences in celebrity worship [2, 22], while several researchers have mentioned that celebrity worship was more prevalent among women than men in the Chinese context [11, 23], which required further research to determine. Based on the views mentioned above, the following hypotheses were posited:

- Hypothesis 1. Social anxiety is positively related to celebrity worship and can predict celebrity worship.
- Hypothesis 2. Females score higher on total CAS scores than males.

### Mobile phone dependence as a mediator

Smartphones provide a wide range of functions and applications (apps), which leads to a small minority of individuals showing symptoms of mobile phone addiction [24]. Several studies have shown that individuals with high levels of social anxiety are more likely to use mobile phones and have higher levels of mobile phone addiction [25–27]. For socially anxious people, activities on their phones can reduce social cues and increase comfort, thus relieving interpersonal stress [28–30]. In addition, social anxiety is a predictor of mobile phone dependence [31–34].

Zsila et al. [8] claimed that problematic social media use correlates with celebrity worship. The existence of various social apps on mobile phones makes it possible to communicate between fans and celebrities [35, 36]. With the popularity of social media and mobile phones, the endless search for celebrity information combined with an unhealthy psychological state may lead to an increase in celebrity worship [37]. As a carrier of social media, over-dependence on mobile phones may also lead to celebrity worship. Despite limited research, we have hypothesized:

- Hypothesis 3. Mobile phone dependence mediates the relationship between social anxiety and celebrity worship.

### Socioeconomic status (SES) as a moderator

Based on ecological systems theory [38], the development of problem behaviors can be considered to be the result of an interaction between environmental factors and an individual’s intrinsic characteristics. Family socioeconomic status (SES) is often measured by parental income, occupation, and education [39, 40]. Low SES families often fail to provide a better environment for their children due to resource constraints and pressures. As a result, their children may experience higher levels of addictive behavior. Previous studies have shown that individuals with low socioeconomic status have higher rates of Internet addiction [41, 42]. Other studies have found that adolescents with high socioeconomic status have a lower risk of social network addiction [28, 43]. However, as a carrier of the Internet and social networks, few studies have explored the influence of family socioeconomic status on mobile phone dependence.

Inconsistent findings have been reported regarding the relationship between celebrity worship and family socioeconomic status. For instance, McCutcheon et al. [10] found that celebrity worship was not associated with SES, while, Cheung and Yeu [23] found that teen idol aliases were associated with lower socioeconomic status. Therefore, whether the impact of SES on celebrity worship needs to be further verified, and we hypothesized:

- Hypothesis 4. SES can predict celebrity worship and moderate the relationship between social anxiety and mobile phone dependence.

### The current study

**Fig. 1:**
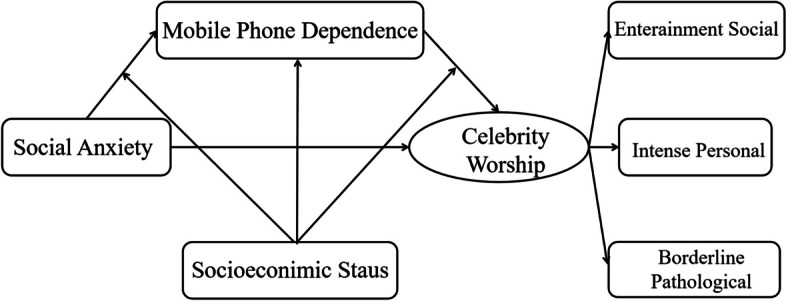
Theoretical model of relations between social anxiety and celebrity worship and mobile phone dependence, SES as a moderator

Based on previous studies, we proposed a moderated mediation model to examine the underlying mechanism of the relationship between social dysfunction and celebrity worship (Fig. 1). Therefore, we proposed three hypotheses: (1) there is a positive correlation between social anxiety and worship; (2) Mobile phone dependence mediates the relationship between social anxiety and celebrity worship; (3) Family socioeconomic status as measured by income, plays a moderating role among social anxiety, mobile phone dependence and celebrity worship (Fig. 1). Furthermore, the difference between females and males on celebrity worship was explored in Chinese sample.

## Methods

### Participants

Participants were recruited from Chinese universities by random sampling. The online assessment was conducted by using the application called “Questionnaire Star”. The “Questionnaire Star” is an online questionnaire platform which can generate an OR code linked to the online questionnaire, delivering it to participants through other social media platforms (e.g., WeChat, Weibo) to obtain their responses. A total of 1,208 people between 19 and 26 years of age participated in the study. After excluding participants who did not complete all questionnaire items and were not college students, data for 1,147 subjects were available for subsequent analysis. The mean age of participants was 20.60 (SD = 1.26) years and 636 were females, 511 were males. Most of them (97.99%) were undergraduates, only a few (2.01%) were graduate students. Besides, all of participants reported that they had a favor celebrity.

### Measures

Data regarding demographic characteristics were collected from participants including information about gender, age, educational level, and family socioeconomic status (SES). The anonymous questionnaires were completed online during May - June 2022 and included:

#### Celebrity worship

Participants’ dedication to their favorite celebrity was assessed by using the 23-item Celebrity Attitude Scale [3, 10, 37], which consists of three sub-scales: Entertainment-Social (ten items; e.g., “Keeping up with news about my favorite celebrity is an entertaining pastime”; (Cronbach’α = 0.885), Intense-Personal (nine items; e.g., “I am obsessed by details of my favorite celebrity’s life”; Cronbach’α = 0.860), and Borderline-Pathological (four items; e.g., “I often feel compelled to learn the personal habits of my favorite celebrity”; Cronbach’α = 0.669). Items are rated on a 5-point Likert scale ranging from 1 (strongly disagree) to 5 (strongly agree). Cronbach’α of the total scale was 0.935 in the present study.

#### Social anxiety

The Liebowitz Social Anxiety Scale (LSAS) was used to assess social anxiety [44]. Participants were asked to self-report their levels of anxiety over the past week. LSAS includes 11 items relating to social situations (e.g., “Talking to people in authority”; Cronbach’α = 0.814) and 13 items relating to operational situations (e.g., “Telephoning in public”; Cronbach’α = 0.752), respectively, evaluating fear and anxiety (subjective experience) and avoidance (objective fact). The assessment time was in the last one week. The Chinese version of the CAS has well-established validity and reliability [45]. The Cronbach’α coefficient of this scale in this study was 0.886.

#### Mobile phone dependence

The mobile phone addiction index scale (MPAI) was used to assess participants’ levels of mobile phone addiction [46]. It contained 17 questions, divided into 4 dimensions, namely inability to control craving (7 items; e.g., “Your friends and family complained about your use of the mobile phone”; Cronbach’α = 0.862), feeling anxious and lost (5 items; e.g., “When out of range for some time, you become preoccupied with the thought of missing a call”; Cronbach’α = 0.838), withdrawal/escape (3 items; e.g., “You have used your mobile phone to talk to others when you were feeling isolated”; Cronbach’α = 0.721), and productivity loss (2 items; e.g., “You find yourself occupied on your mobile phone when you should be doing other things, and it causes a problem”; Cronbach’α = 0.683). Items are rated on a 5-pt Likert scale from 1 = *Never* to 5 = *Very often*. The scale includes 17 questions that are answered using a five-point scale, and higher scores indicate greater mobile phone dependence (Cronbach’α = 0.932 in the current study). In addition, eight of the questions screen for phone addiction, with a score of 3 or greater, indicating mobile phone addiction.

#### Family socioeconomic status

Parental income level was used as a proxy variable for socioeconomic status. Parental income levels were measured on a seven-point scale: 1 = lower than 2000, 2 = 2000 to 3000, 3 = 3000 to 5000, 4 = 5000 to 8000, 5 = 8000 to 15,000, 6 = 15,000 to 30,000, 7 = higher than 30,000 Chinese Renminbi (RMB) per month. In China, a monthly income of less than 3,000 belongs to a low socioeconomic status family, while a monthly income of more than 15,000 belongs to a high socioeconomic status family.

### Statistical analysis

We used SPSS to calculate the correlation coefficients between each variable. Independent sample t-tests were used to assess the influence of gender on celebrity worship. Linear regression was used to assess differences in celebrity worship by age. Then, structural equation model using Mplus 17.0 was used to explore mediating and moderating effects [47, 48]. Indirect effects were tested using the 5000 bootstrap technique [49], and confidence intervals (95% CI) that did not contain zero indicated significant indirect effects [50]. The extent of possible bias was tested statistically by use of Harman’s single factor analysis [51].

## Results

### Test of common method bias

To reduce the bias from the subjects’ self-reports, polygraph tests and anonymous surveys were conducted. Results of the Harman’s test showed that the characteristic root of 10 factors was greater than 1, and the explanation rate of the first factor was 19.99%, far less than the critical value of 40%. Thus, no serious common method bias was detected.

### Preliminary analyses

In Table 1, results of the correlational analysis revealed significant correlations between SES and CAS total scores, and the three CAS sub-scales scores: entertainment-social (*r*_*p*_ = 0.95, *p* < .001), intense-personal (*r*_*p*_ = 0.95, *p* < .001) and borderline-pathological dimension (*r*_*p*_ = 0.74, *p* < .001). Mobile phone use was also found to be significantly associated with social anxiety (*r*_*p*_ = 0.30, *p* < .001) and celebrity worship (*r*_*p*_ = 0.19, *p* < .001). Therefore, subsequent mediation analysis was performed. Through independent sample t-tests, there was a significant difference between male and female in the entertainment-social dimension (Table 2). Besides, the multicollinearity test was conducted, and results showed that the variance inflation factor (VIF) of each variable was less than 5, which meaning that there was no multicollinearity problem [52].

**Table 1:** Correlation matrix of Celebrity worship, Social anxiety, Mobile Phone Use, Emptiness and SES

	M	SD	1	2	3	4	5	6	7
1. CAS total	76.72	15.67	1						
2. Entertainment Social	35.86	7.37	0.954***	1					
3. Intense Personal	31.16	6.85	0.952***	0.855***	1				
4. Borderline-Pathological	9.70	2.85	0.739***	0.604***	0.621***	1			
5. Social Anxiety	62.97	9.98	0.389***	0.334***	0.352***	0.429***	1		
6. MPAI	52.23	13.02	0.187***	0.152***	0.192***	0.173***	0.304***	1	
7. SES	4.23	1.27	0.178***	0.191***	0.197***	0.013	0.034	0.037	1

*CAS* celebrity attitude scale, *MPAI* mobile phone addiction index scale, *SES* family socioeconomic status

***p<0.001, after control sex and age

**Table 2:** Differences between females and males in analyzed variables

	Female (*N* = 636)		Male (*N* = 511)		t	*p*
	M	SD	M	SD		
Celebrity Worship	77.48	15.41	75.76	15.94	1.85	0.065
Entertainment-Social subscale	32.91	6.66	31.96	6.82	2.37	0.018
Intense-Personal subscale	31.39	6.70	30.89	7.03	1.22	0.224
Borderline-Pathological subscale	13.33	3.52	13.07	3.43	1.24	0.215
Social Anxiety	62.75	10.13	63.24	9.80	− 0.82	0.411
Mobile Phone Dependence	52.58	13.00	51.80	13.04	1.00	0.313

### Mediating Effect of Mobile phone dependence

This study used social anxiety as the independent variable, celebrity worship as the dependent variable, mobile phone dependence as the mediating variable, and SES as the moderating variable. Besides, all of these variables were centralized. Taking age, celebrity type and gender as control variables, the mediating effect was analyzed by using the SPSS macro program PROCESS v3.4.1 compiled by Hayes (2013).

 In the mediation model (Table 3), mobile phone dependence significantly mediated the association between social anxiety and celebrity worship as shown in Tables 3 and 95%CI = [0.006, 0.083]. In addition, social anxiety positively predicted mobile phone dependence (*β =* 0.31, *SE =* 0.04, *p <* .001), and mobile phone dependence positively predicted celebrity worship (*β* = 0.10, *SE =* 0.04, *p* < .01).

**Table 3:** Results of the mediation model

Dependent variable	Celebrity worship			Mobile phone addiction			Celebrity worship		
	*β*	*SE*	*t*	*β*	*SE*	*t*	*β*	*SE*	*t*
Social anxiety	0.39	0.05	12.50^***^	0.31	0.04	9.43^***^	0.36	0.05	11.06^***^
Mobile phone addiction							0.10	0.04	2.79^**^
*R*^*2*^	0.20			0.11			0.21		
*F*	41.92^***^			20.71^***^			36.51^***^		

^**^p < 0. 01, ^***^ *p*  < 0. 001

### Moderating effect of SES

In Table 4, the interaction of social anxiety and SES cannot positively predict celebrity worship (*β =* 0.03, *SE =* 0.04, *p > .*05). However, this interaction can negatively predict mobile phone dependence (*β =* − 0.11, *SE =* 0.03, *p <* .01). Besides, SES also did not moderate the link between mobile phone dependence and celebrity worship (*β* =−0.03, *SE =* 0.03, *p >* .05).

**Table 4:** Results of the moderated mediation model

		*β*	*SE*	*t*	95%CI		*R*^*2*^	*F*
					LLCI	ULCI		
Mobile phone addiction	Social Anxiety	0.41	0.04	9.59^***^	0.32	0.49	0.13	17.30^***^
	SES	0.58	0.35	1.68	− 0.10	1.26		
	Social Anxiety × SES	− 0.11	0.03	1.68^**^	− 0.17	− 0.05		
Celebrity Worship	Social Anxiety	0.52	0.05	10.70^***^	0.42	0.61	0.23	27.37^***^
	Mobile phone addiction	0.10	0.04	1.68^**^	0.03	0.18		
	SES	1.70	0.36	4.67^***^	0.99	2.42		
	Social Anxiety × SES	0.03	0.04	0.81	− 0.04	0.10		
	Mobile phone addiction ×SES	− 0.03	0.03	− 0.84	− 0.09	0.04		

^**^ *p* < 0. 01, ^***^ *p* < 0. 001

**Fig. 2:**
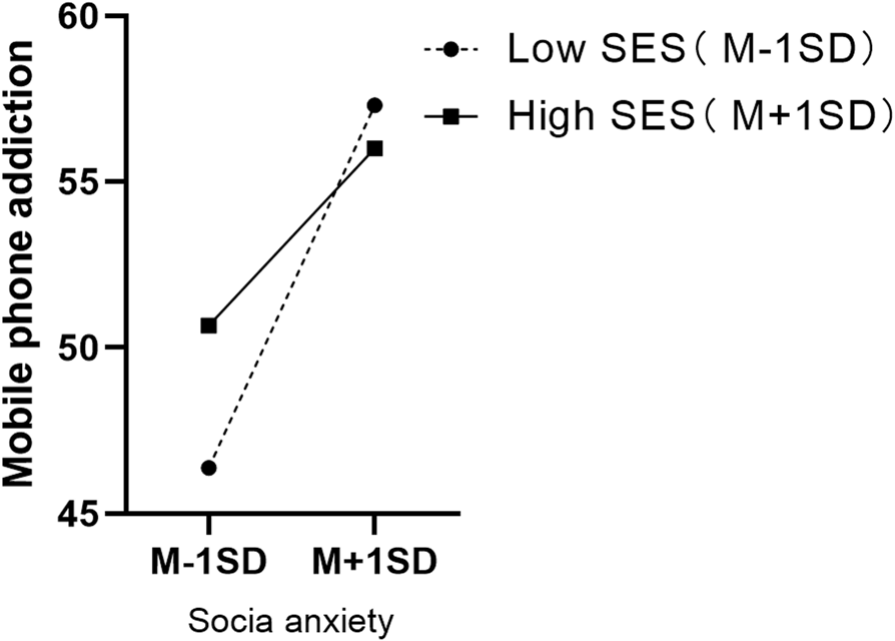
Simple slopes in high and low levels of SES. Low meaning in life=−1, High meaning in life = 1

**Fig. 3:**
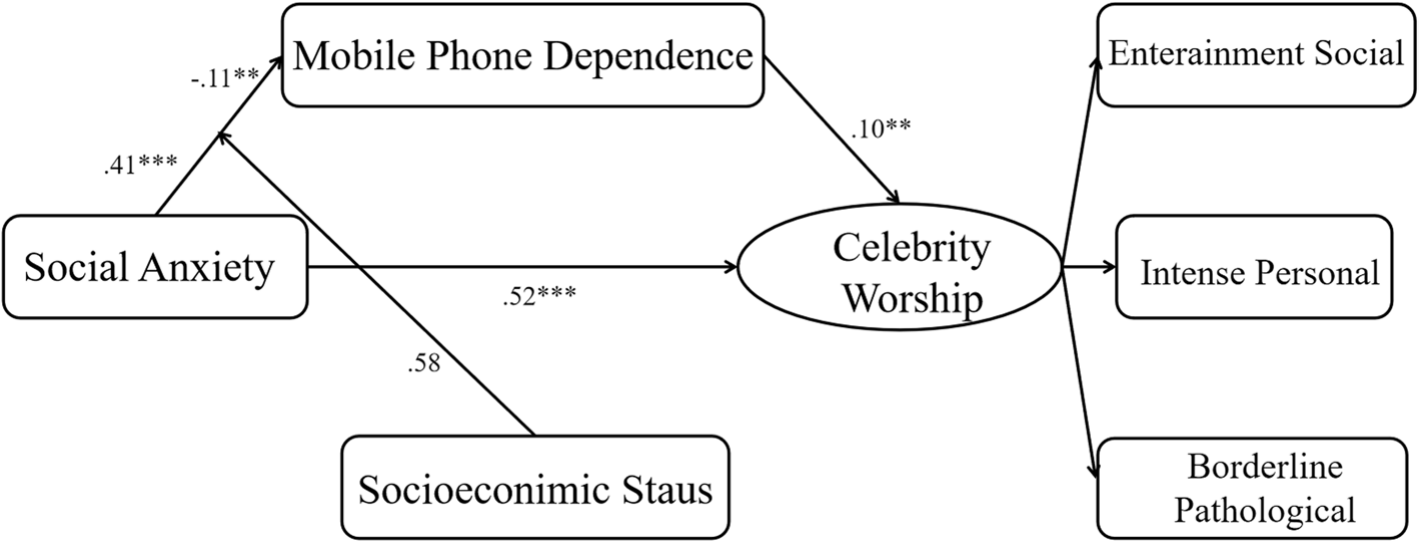
The final moderated mediation model plot

To further explore the interaction between social anxiety and SES, the simple slope tests were conducted to illustrate how social anxiety was associated with mobile phone dependence in low and high levels (M ± SD) of SES. According to Fig. 2, when SES level is low, social anxiety has a greater predictive effect on mobile phone dependence (1 SD below; *b*_*simple*_ = 0.78, *t* = 8.32, *p* < .001). At a high level of SES, individuals with high social anxiety have lower mobile phone dependence scores. Figure 3 displayed the significant interactive effect of SES in buffering the effect of mobile phone dependence on celebrity worship.

## Discussion

Previous studies have focused solely on the relationship between an unhealthy psychological state and celebrity worship, with little exploration of the underlying mechanisms. This study is the first to explore the potential impact of social anxiety on celebrity worship. A moderated mediation model was constructed according to the Absorption-addiction model and the social compensation hypothesis. Our results suggest that social anxiety influences celebrity worship through mobile phone dependence, while SES is found to moderate the impact of social anxiety on mobile phone dependence.

There is a significant positive correlation between social anxiety and celebrity worship, indicating that young people with high social anxiety have a higher degree of celebrity infatuation. This finding supports H1. The Absorption-addiction model suggests that an introverted nature and lack of meaningful relationships in celebrity worshipper are more likely to celebrity worship [3]. Mairet et al. [53], Stemberger et al. [54] and Zhong et al. [55] have proposed that individuals with social anxiety tend to exhibit introverted tendencies and sensitive to interpersonal relationships, which may lead to their addiction to idols.

Consistent with previous entertainment-social dimension studies, we did not find a gender difference on celebrity worship [2, 3]. However, in the entertainment-social dimension, women score higher than men, suggesting that women are more inclined to focus on celebrities, such as engaging in activities like watching, reading, or listening to news about their favorite celebrities for entertainment purposes [56]. The results obtained in the context of Chinese culture are also consistent with the results obtained in a sample of Philippines [57, 58], which partially supports H2.

The present study showed that mobile phone dependence mediated the relationship between social anxiety and celebrity worship, verifying H3. Social anxiety leads to mobile phone dependence, and then leads to star-chasing behavior, and is consistent with previous studies that have shown that social anxiety and phone dependence go hand in hand [31–34]. Elhai et al. [59] have shown that socially anxious youth may seek comfort by checking messages, which can help relieve anxiety and creates dependence on mobile phones. Prolonged use of mobile phones can increase the dependence on social media [60, 61] and browsing for relevant celebrity information, may contribute to excessive attention on celebrities.

Contrary to previous studies [22, 23], we found a positive relationship between family socioeconomic status and celebrity worship. Individuals who have high family economic status have more financial support to maintain parasocial relationships with celebrities [62]. For example, peripheral products for celebrities, gifts given to celebrities, and close encounter (requires expenditure) can all improve the pleasure gained by individuals’ infatuation with celebrities, thus increasing celebrity worship.

In addition, SES played a regulatory role in the first half of the mediation path. Youth with high family socioeconomic status may be less mobile phone dependent and thus less prone to celebrity worship. It has been shown that parents with high SES have higher parental involvement [63, 64] and more positive parenting [65], which can improve youths’ cognitive ability and assist them to develop good habits, thus reducing mobile phone dependence. However, SES did not moderate the effects of social anxiety on celebrity worship, and phone dependence on celebrity worship. Therefore, H4 was partially verified. The reason may be that SES was unable to exert influence after the formation of mobile phone dependence. In addition, as distant factors for celebrity worship, social anxiety and SES have a weak influence [66]. Therefore, the influence of celebrity worship may be influenced by other proximal factors. When it comes to interventions for youth and teenagers from low-income families, fostering peer communication and companionship could be a viable approach to reduce social anxiety and mobile phone dependence.

### Limitations and future directions

There are several limitations of this study that require acknowledgment. First, the cross-sectional design of this study was not able to examine the causal relationship between social anxiety and celebrity worship. Second, we only used distant factors purported to affect celebrity worship, thus, we were unable to accurately explore other mechanisms that might affect fans’ culture. In the future, longitudinal studies are required to investigate the relationship between psychologically unhealthy individuals and celebrity worship. In addition, studies of parasocial relationships, self-identity, social media use and other factors are also required to explore the formation of fans’ culture. Third, the age bracket of participants in this study was between 19 and 26 years, and most of the participants are college students. Therefore, it is necessary to expand the age range in the future study to explore the development trajectory of celebrity worship.

## Conclusion

This study extends the model of Absorption-addiction to show that social anxiety increases the risk of celebrity worship. At the same time, social anxiety and SES have an interaction effect on celebrity worship through mobile phone dependence, suggesting that higher SES may relieve the effects of social anxiety on mobile phone dependence.

## Data Availability

Codes used in this study are available from the authors QY and ZW upon request.
